# Patterns of functional enzyme activity in fungus farming ambrosia beetles

**DOI:** 10.1186/1742-9994-9-13

**Published:** 2012-06-06

**Authors:** Henrik H De Fine Licht, Peter H W Biedermann

**Affiliations:** 1 Microbial Ecology Group, Department of Biology, Lund University, Ecology Building, Solvegatan 37, SE-22362, Lund, Sweden; 2 Department of Behavioural Ecology, Institute of Ecology & Evolution, University of Bern, Baltzerstrasse 6, CH-3012, Bern, Switzerland

**Keywords:** Symbiosis, Digestion, Enzyme, Insoluble chromogenic substrates, Xylomycetophagy, *Xyleborinus saxesenii*, Insect fungus farming, Social evolution, Division of labor

## Abstract

**Introduction:**

In wood-dwelling fungus-farming weevils, the so-called ambrosia beetles (Curculionidae: Scolytinae and Platypodinae), wood in the excavated tunnels is used as a medium for cultivating fungi by the combined action of digging larvae (which create more space for the fungi to grow) and of adults sowing and pruning the fungus. The beetles are obligately dependent on the fungus that provides essential vitamins, amino acids and sterols. However, to what extent microbial enzymes support fungus farming in ambrosia beetles is unknown. Here we measure (i) 13 plant cell-wall degrading enzymes in the fungus garden microbial consortium of the ambrosia beetle *Xyleborinus saxesenii*, including its primary fungal symbionts, in three compartments of laboratory maintained nests, at different time points after gallery foundation and (ii) four specific enzymes that may be either insect or microbially derived in *X. saxesenii* adult and larval individuals.

**Results:**

We discovered that the activity of cellulases in ambrosia fungus gardens is relatively small compared to the activities of other cellulolytic enzymes. Enzyme activity in all compartments of the garden was mainly directed towards hemicellulose carbohydrates such as xylan, glucomannan and callose. Hemicellulolytic enzyme activity within the brood chamber increased with gallery age, whereas irrespective of the age of the gallery, the highest overall enzyme activity were detected in the gallery dump material expelled by the beetles. Interestingly *endo*-β-1,3(4)-glucanase activity capable of callose degradation was identified in whole-body extracts of both larvae and adult *X. saxesenii*, whereas *endo*-β-1,4-xylanase activity was exclusively detected in larvae.

**Conclusion:**

Similar to closely related fungi associated with bark beetles in phloem, the microbial symbionts of ambrosia beetles hardly degrade cellulose. Instead, their enzyme activity is directed mainly towards comparatively more easily accessible hemicellulose components of the ray-parenchyma cells in the wood xylem. Furthermore, the detection of xylanolytic enzymes exclusively in larvae (which feed on fungus colonized wood) and not in adults (which feed only on fungi) indicates that only larvae (pre-) digest plant cell wall structures. This implies that in *X. saxesenii* and likely also in many other ambrosia beetles, adults and larvae do not compete for the same food within their nests - in contrast, larvae increase colony fitness by facilitating enzymatic wood degradation and fungus cultivation.

## Introduction

Insects are the most abundant and diverse animal class on earth [1]. A key factor for their enormous success are adaptations to novel environments and food sources with the help of symbiotic microorganisms [2]. Insect hosts maintain prokaryotic, fungal, and bacterial associates in a variety of ways, which help them in nutrient acquisition and recycling, environmental detoxification, and defense against antagonists. By means of microbial symbionts insects are able (i) to detoxify toxic metabolites and (ii) to produce nutrients from plant material low in insect-accessible molecules, despite the plant material often being rich in structural polysaccharides (cross-linking glycans, cellulose and lignin) [3]. In most instances this occurs internally inside the insect host with the aid of an abundant microbial gut flora [4-6], however, there are several notable exceptions of insect lineages that cultivate microbes externally on plant material [7,8]. These ectosymbionts are either kept in “gardens” and consumed directly by their insect hosts (e.g. certain lineages of fungus-growing ants, termites, and ambrosia beetles; [9]), or contribute indirectly by increasing the nutrient content of the diet (e.g. bark beetles that feed on fungus infested phloem; [10]), by degradation of toxic plant compounds (e.g. terpenes by bark beetle associated fungi; [11,12]), or by provisioning of extracellular enzymes that facilitate wood ingestion or wood burrowing (e.g. wood wasps; [5,13]).

External symbionts of insects are typically filamentous fungi and associated yeasts and bacteria that may be transported in concert in mycetangia (also termed mycangia [14]), which are specialized organs primarily for fungal spore transmission that ensure successful re-establishment of the nutritional symbiosis after dispersal. Mycetangia have evolved independently in many different fungus associated insects such as wood and phloem feeding beetles, gall midges and wood wasps [15-17]. The active care and maintenance of the fungal crops by the insect hosts after dispersal is, however, rare. Only three insect lineages, notably the fungus-growing ants, termites and ambrosia beetles, are true fungus farmers. Within task sharing societies they not only propagate, but also actively cultivate and sustainably harvest microbial gardens without exhausting their crops across one or more offspring generations (i.e. advanced fungiculture; [9]).

Ambrosia beetle is an ecological term used for all weevils that farm fungi within tunnel systems (galleries) in the xylem (= wood) of trees. Ambrosia farming is only found in Scolytinae and Platypodinae and evolved repeatedly at least nine times from the phloem feeding habit without any known reversal to non-farming [18,19]. Female ambrosia beetles seek out recently dead trees where they bore into the xylem and initiate nest building by laying eggs and inoculating tunnel walls with mutualistic fungi. When larvae emerge they feed on the fungus and in some species further expand the gallery [20]. Depending on the species and environmental conditions adults repeat this cycle by either dispersing immediately following pupation or remain in their natal gallery and engage in cooperative breeding for more generations before dispersing [9,21].

The relationship between ambrosia beetles and their fungi is often species (or genus) specific, with highly selective transmission of the primary symbionts in mycetangia by dispersing beetles [20,22]. These so-called ambrosia fungi (usually species of the ascomycete genera *Ambrosiella* and *Raffaelea*) form layers of conidiophores on the tunnel walls that produce nutrient rich conidiospores for larval and adult beetle nutrition. Secondary symbionts, such as other filamentous fungi (e.g. *Fusarium*, *Graphium*, *Ophiostoma*, *Paecilomyces*, *Penicillium*[23,24]), yeasts (e.g. *Candida*[25]) and bacteria [26,27], are also present within galleries and often passively vectored in small amounts attached to the integuments of dispersing females [15,20]. However, the primary mutualistic ambrosia fungus is known for only a minority of the 3000 species worldwide [28-30], and there has only been a single attempt to characterize the entire microbiome of an ambrosia gallery [31].

Studies on the dynamics of filamentous fungi in xyleborine ambrosia beetle galleries [23,24], suggest that propagates of mutualistic ambrosia fungi (*Ambrosiella* and *Raffaelea*) are passively spread on tunnel walls from the mycetangia or via beetle feces during the excavation by the gallery founding female. This ensures that the mutualistic fungi dominate the gallery microbial flora initially while eggs are laid and larvae develop. Later, when the first offspring mature, other saprobic fungi (secondary symbionts like *Penicillium* and *Paecilomyces*) start to appear and increase in frequency over time. These opportunistic fungi dominate the microbial gallery flora at the time when the gallery is abandoned and all individuals disperse to found new galleries [23]. The secondary symbionts also dominate in the gallery dumps of our study species, the ambrosia beetle *Xyleborinus saxesenii* (Fruit-tree pinhole borer); they are relatively rare in freshly excavated parts of the brood chamber and their presence negatively affects larval numbers [24].

Larvae of *X. saxesenii* do not only feed on ambrosia fungi, like the adults and larvae of many other ambrosia beetles, but feed xylomycetophagously (i.e. feeding on fungus infested wood) [32]. In this way they (a) create more space for the developing fungus to form conidiophores on the gallery walls, (b) lower competition between group members by enlargement of the nest space, (c), likely reduce the growth of unidentified molds, possibly by gregariously feeding on them [21], and (d) fertilize the growing ambrosia fungus with the finely fragmented woody sawdust in their feces [21,33]. This apparently allows *X. saxesenii,* and probably other xylomycetophagous ambrosia beetle species, to establish galleries for several consecutive offspring generations. However, nothing is known about the mechanism or the enzymatic machinery whereby these beetles together with the consortium of symbiotic fungi utilize the surrounding wood.

In contrast to xylem dwelling ambrosia beetles, weevils dwelling in the inner bark phloem and feeding phloeophagously (phloem feeding) or phloeomycetophagously (feeding on fungus infested phloem) are termed bark beetles [20]. Their primary associates, the ophiostomatoid fungi, are close relatives of ambrosia fungi and are known to produce a variety of hemicellulolytic enzymes [34-38], although *Ophiostoma* fungi in general leave the cellulose and cross-linking glycans mostly intact and instead utilize storage products in the living ray parenchyma [29]. The beetles and internal gut microbes may also contribute enzymes as larvae of a bark beetle (*Phloeosinus bicolor*) showed α-amylase-, invertase-, maltase-, lactase-, and protease-activities together with some hydrolytic activity on a substrate of hemicellulose but not on cellulose [39]. Similarly, in adults of the phloem feeding *Ips cembrae* consistent activity against hemicellulose together with pectinase-, α-glucosidase-, β-glucosidase-, α-galactosidase-, β-galactosidase-, trehalase-, serine protease-, peptidase-, and lipase-activities were detected in the intestinal lumen [40]. In general, the bark beetle associated fungi (e.g. the genera *Ceratosytiopsis, Entomocorticium, Grosmannia* and *Ophiostoma*[41,42]), in addition to associated yeasts [12,41,43], and bacteria [44,45] are capable of producing a variety of enzymes catalyzing (a) protein/peptide degradation (endo-, exoproteases and peptidases), (b) polysaccharide/starch/sugar degradation (glycoside-hydrolytic enzymes) and (c) fat/fatty acid degradation (lipases) [34-36,41,42,46-49].

Here the activity of the major groups of plant cell-wall degrading enzymes: cellulases, hemicellulases, pectinases, in addition to proteases and α-amylases in the ambrosia beetle system are investigated for the first time. We take advantage of a recently developed method to maintain ambrosia beetle galleries of *X. saxesenii* for consecutive generations *in-vitro* in the laboratory [50]. We show that ambrosia beetles and their associated microbiome mainly degrade the hemicellulose component of xylem wood in addition to more readily degradable simple sugars. Furthermore, we document that larvae and adults possess different enzyme profiles, which adds an additional layer of complexity to the division of behavioral tasks between life-stages already reported within the highly social societies of *X. saxesenii*[21].

## Results

### Gallery enzyme activity

**Figure 1:**
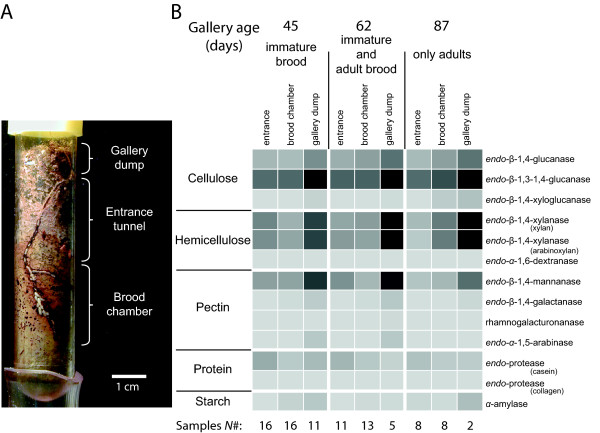
**Glycoside hydrolytic enzyme activity of*X. saxesenii *ambrosia beetle galleries. A.** Picture of a *X. saxesenii* gallery in artificial media around day 45 after gallery foundation. Note the three distinct compartments, where samples for enzyme activity measurements were collected: entrance tunnel, brood chamber, and gallery dump. Many white larvae and a few light brown teneral females are visible in the brood chamber and the lower part of the entrance tunnel. **B.** Enzyme activity of 13 specific carbohydrate active enzymes presented as a heatmap with darker coloration showing higher enzyme activity. Enzyme activity was measured when only immature brood was present, when both immature and adult brood were present, and finally when only adult brood were present (45, 62 and 87 days respectively, after gallery foundation by a single mated female). Enzymes are divided into functional groups according to the plant cell structure functioning as substrate for enzymatic hydrolysis.

We measured enzyme activity of samples taken at three time points after gallery foundation in all three gallery compartments (Figure 1A): (1) gallery dump samples, containing all the waste-material (sawdust, feces, fungus) that is shuffled out of the entrance tunnel by the adult females, (2) samples of the fungus infested substrate from the walls of the entrance tunnel, which is the oldest part of the nest and vertically penetrates the substrate, and (3) samples of the fungus infested substrate of the brood chamber, where the major part of the mutualistic fungus is growing and the brood is developing. Six specific enzyme activities (endo-β-1,4-glucanase, endo-β-1,3(4)-glucanase, endo-β-1,4-xylanase (xylan and arabinoxylan), endo-β-1,4-mannanase, and endo-protease (casein)) were consistently detected in all gallery samples when using 13 different enzymatic substrates (Figure 1B, [Additional file 1: Figure S1]). Enzyme activities varied significantly between the three gallery compartments (log-likelihood ANOVA comparison of final mixed models with reduced null models: likelihood-ratio_3,5_ = 14.1 – 50.4, *p* = < 0.0001 – 0.0009), but were not significantly influenced by the number of larvae and adults present in the gallery at the time of wall-material sampling (log-likelihood ANOVA comparison of final mixed models with reduced null models: likelihood-ratio_5,11_ = 2.0 – 8.7, *p* = 0.1884 – 0.9169). The plant cell-wall degrading cellulases, endo-xylanases and pectinases had a consistently higher activity in the gallery dump material compared to the entrance tunnel and the brood chamber (Figure 1B, [Additional file 1: Figure S1]), whereas endo-protease activity against casein showed the opposite trend with the highest enzyme activity in the entrance tunnel (Figure 1B, [Additional file 1: S1]). The increased enzyme activity of plant cell-wall degrading enzymes in the gallery dump was also evident from the partial least square regression analysis because these specific enzymes correlated (i.e. clustered) more closely to the gallery dump than both the entrance tunnel and the brood chamber [see Additional file 1].

Cellulolytic activity was similar between the entrance tunnel and brood chamber across gallery ages, whereas *endo-*β-1,4-xylanase (xylan and arabinoxylan) and *endo-*β-1,4-mannanase activity changed across age cohorts most notably with an increase in enzyme activity in the gallery dump over time (log-likelihood ANOVA comparison of final mixed models with reduced null models: likelihood-ratio_5,11_ = 12.7 – 16.9, *p* = 0.0095 – 0.0472, Figure 1B). For these three enzymes we also noted a consistent but non-significant trend of higher activity in the entrance tunnel compared to the brood chamber at *age45* (i.e., 45 days after gallery foundation), similar activity at *age62* and the opposite pattern at *age87* [see Additional file 1].

Enzyme activity against the substrates xyloglucan, galactan, rhamnogalacturonan, debranched arabinan and amylose tended to be highest in the gallery dump [see Additional file 1]. Because these enzyme activities were only sporadically detected, we analyzed each age cohort separately using a non-parametric Kruskal-Wallis test [see Additional file 1]. Enzyme activities against the substrates dextran and collagen were not detected in any sample (Figure 1B).

### Adult and larvae enzyme activity

**Figure 2:**
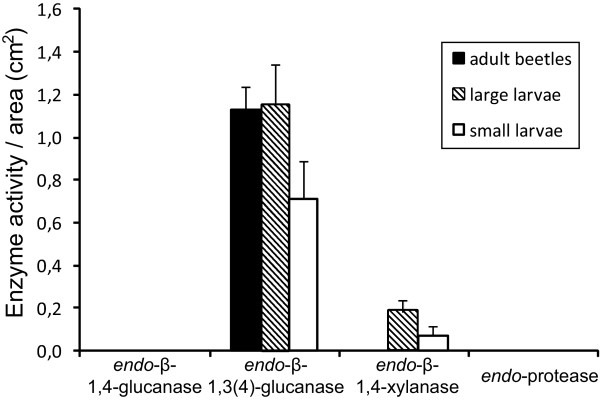
***Endo-*β-1,4-glucanase, *endo-*β-1,3(4)-glucanase, *endo-*β-1,4-xylanse and *endo*-protease activity (mean area of blue coloration in AZCL-plate assays + SE) of adult (*n*** **= 14 × 3 adults), large (2**^**nd**^**/3**^**rd **^**instar, *n*** **= 14 × 4 larvae) and small (1**^**st **^**instar, *n*** **= 8 × 12 larvae)* X. saxesenii *larvae, respectively. ***Endo-*β-1,3(4)-glucanase activity is present in all three life stages, whereas *endo-*β-1,4-xylanase activity is not present in adult beetles but only detected in large and small larvae.

*Endo-*β-1,3(4)-glucanase (beta-glucan) activity was detected in 1^st^, 2^nd^/3^rd^ instar larvae and adults (Figure 2), whereas *endo-*β-1,4-xylanase activity was detected in 1^st^ to 3^rd^ instar larvae with highest activity during 2^nd^ and 3^rd^ instars, but not in adult beetles (Figure 2). No statistical analysis was performed on enzyme activities extracted from larvae or beetles because although samples were approximately standardized to the same total biomass the inherent physiological difference between larvae and adult would render the result ambiguous. No *endo-*β-1,4-glucanase or endo-protease (casein) activity were detected in adults or larvae (Figure 2).

## Discussion

### Gallery enzyme activity

Plant cell-wall degrading cellulases, *endo-*xylanases and the pectinolytic *endo-*β-1,4-mannanase dominate the enzymatic profile but also consistent *endo-*protease activity against casein were detected at all measured time-points in all three gallery compartments (Figure 1B). Taken together the enzymatic profile of the microbial consortium of *X. saxesenii* ambrosia galleries resembles that of common saprotrophic ascomycete and basidiomycete fungi [51-53], highlighting the universal similarity of enzymes required in the initial degradation of recently dead wood material. The production of extracellular enzymes by filamentous fungi is highly dependent on the growth medium and external conditions such as temperature and moisture etc. [51]. Hence it is extremely difficult if not impossible to obtain natural enzyme activity profiles under *in-vitro* laboratory conditions, as the actual micro-habitat experienced by microbes in nature cannot be fully replicated. The relatively high *endo*-protease and possibly also α-amylase activity detected in our samples (Figure 1B), for example, is most likely because of casein and starch used in the artificial breeding medium and does not reflect the natural situation. Despite this caveat, the detailed enzymatic measurements of laboratory maintained and age-controlled beetle galleries containing all the naturally vectored symbiotic microbes provide an informative substitute for natural measurements of the highly inaccessible ambrosia beetle galleries deep inside wood.

*Endo-*xylanase activity increased in the brood chamber, but decreased in the entrance tunnel with gallery age ([Additional file 1: Figure S1]). In addition, when comparing the *endo-*β-1,4-glucanase and *endo-*β-1,4-mannanase activity between compartments within galleries, samples from the entrance tunnel and brood chamber showed remarkably similar enzymatic profiles, whereas gallery dump samples had much higher activities (Figure 1B). These changes in enzymatic activity most likely reflected progression in the degradation of the wood substrate surrounding the galleries. Only cellulase activity was remarkably similar and low in all gallery compartments at all age stages (Figure 1B). Unfortunately, we are unable to distinguish whether these shifts in enzyme activity are due to changes (i) in beetle activities, (ii) in *endo-*xylanase production by the resident microbes, or (iii) in the succession of microbes in the galleries. Ambrosia beetle galleries are not static environments and conditions (e.g. humidity, degradation) and the composition of the associated microbial consortium changes both between gallery compartments and as tunnel parts age. Ambrosia fungi, as the primary food source for the beetles, only dominate the microbiome in freshly excavated gallery parts where the brood develop and are later replaced by secondary saprobic symbionts that continue degradation [23,24]. Therefore, the observed enzyme activity in the expelled saw-dust material in the gallery dump is likely produced by opportunistic bacteria and fungi not necessarily involved in the nutrition of the insects, as found in certain fungus-growing ant dumps [54]. The expelled saw-dust is probably of little nutritional value and instead represents a source of potential contamination that needs removal [9]. However, this begs the question how the relatively lower enzyme activity of the microbial consortium in the freshly excavated gallery parts is able to sustain ambrosia beetle nutrition? The high hemicellulolytic activity against xylan, glucomannan and callose, but only little activity against cellulose (Figure 1B), show that the gallery microbiome preferentially degrades hemicellulose components of the ray-parenchyma cells in the xylem. This is in contrast to bark beetle microbiomes in the phloem, which apparently leaves almost all of the structural plant cell wall components (cellulose, hemicellulose and pectin) intact [29]. Degradation of hemicellulose components is energetically less costly than complete cellulose degradation [51], however, the reliance on hemicellulases emphasizes that the xylem niche within recently dead wood is transient before the microbiome has to be provisioned with new material either by excavation of tunnel systems or dispersal to new hosts. Indeed, ambrosia beetles leave their galleries with their mutualists stored in mycetangia when opportunistic saprobes invade [23], which may coincide with depletion of the more easily accessible plant cell wall components.

### Adult and larvae enzyme activity

Endosymbionts play a crucial role in nutrient acquisition in many wood-feeding arthropods, like termites or wood-boring beetles [2,4,49]. In bark and ambrosia beetles they seem of minor importance because these beetles feed either on (fungus infested) phloem (i.e. bark beetles) or fungi/fungus infested xylem (i.e. ambrosia beetles). The gut flora of ambrosia beetles has not been studied, but for bark beetles the species richness in larval and adult guts is relatively low [45,55]. The endosymbiotic yeasts and bacteria in bark beetles have been shown to detoxify poisonous wood compounds (e.g. tannins [11]) and fix nitrogen [56]. However, their role for degradation appears rather small compared to the primary fungal ectosymbionts that are growing within the galleries of *Ips* and *Dendroctonus* beetles [45,55]. As most ambrosia beetle species feed solely on fungus tissue, an endogenous production of plant cell wall degrading enzymes either by the beetles or associated endosymbionts in these species is not expected, but this may be different in larvae of *X. saxesenii* and other ambrosia beetles in the genus *Xyleborinus* that ingest both fungal tissue and particles of wood while feeding [21,32]. Indeed, the different nutrition of larvae and adults in *X. saxesenii* was also reflected by *endo-*β-1,4-xylanase activity observed in whole-body extracts of larvae, but not of adults (Figure 2). *Endo-*β-1,4-xylanase enzymes might be produced by gut endosymbionts that are either specific to the larvae (larval specific bacteria are known from *Ips* and *Dendroctonus* bark beetles [55,57]), or endosymbionts are present in both larvae and adults but facultatively produce and secrete *endo-*β-1,4-xylanases depending on context. A microbial origin of these enzymes is possible because insects are rarely capable of producing plant cell-wall degrading enzymes themselves, although an increasing number of putative genes coding for cellulases, hemicellulases and pectinases are being discovered in the genomes of wood dwelling beetles [58], which in certain cases appear to be horizontally acquired from bacteria [59,60]. A few beetle species have been shown to synthesize xylanase endogenously, for example larvae and adults of the wood-boring beetle *Phaedon cochleariae*[61] and the coffee-berry borer *Hypothenemus hampei* [UniProt:E2J6M9]. The latter is a scolytine beetle, and it therefore is possible that the *endo-*β-1,3(4)-glucanases and *endo-*β-1,4-xylanases found in our study may be endogenously produced by *X. saxesenii*.

A third possibility is that larval and adult plant cell-wall degrading enzymes are of ectosymbiotic origin, i.e., they are fungus derived. Enzyme acquisition by feeding on fungi is well known from several fungal-insect mutualisms (c.f. the acquired enzyme hypothesis [13,62], Table 1). Related bark beetles carry yeasts and bacteria in their intestines [43,55,57] and feed on phloem that is often infested by ophiostomatoid fungi [16], which is likely to provide ample opportunity for the acquisition of microbial enzymes. If the *endo*-β-1,4-xylanase found in larvae of *X. saxesenii* is fungus derived, that would either imply that the fungus exclusively produces endo-β-1,4-xylanase in the structures eaten by the larvae and not by the adults (e.g. it is known that enzyme activity of *Ophiostoma* species vary between mycelium and asexual fruiting structures [35]) or that the larvae but not the adults avoid internal proteolysis of this enzyme. Irrespective of enzymatic origin, the breakdown of cross-linking glycans within the larval intestinal tract may (i) have a positive influence on larval nutrition and (ii) could be enhanced by active mixing of small woody particles with fungus derived plant cell-wall degrading enzymes.

**Table 1:** Overview of highly derived, obligate nutritional symbioses between insects and fungi

	**Coleoptera**			**Diptera**	**Hymenoptera**		**Isoptera**
	**Ambrosia beetles**	**Bark beetles^1^**	**Ship-timber beetles**	**Gall midges**	**Wood wasps**	**Fungus-growing ants**	**Fungus-growing termites**
Insect family	Curculionidae	Curculionidae	Lymexylidae	Cecidomyiidae	Xiphydriidae, Orussidae, Anaxyelidae, Siricidae	Formicidae	Termitidae
Mutualistic fungi	Ascomycota (*Ambrosiella, Raffaelea*, *Fusarium*)	Ascomycota (*Ophiostoma, Ceratocystiopsis, Grosmannia*) Basidiomycota *(Entomocorticium*)	Ascomycota (*Endomyces*)	Ascomycota (*Lasioptera, Ramichloridium*)	Basidiomycota (*Cerrena, Stereum*, *Amylostereum*); Ascomycota (*Daldinia decipiens*, *Entonaema cinnabarina*)	Basidiomycota (*Leucocoprinus, Leucoagaricus* and the family Pterulaceae)	Basidiomycota (*Termitomyces*)
Age of symbiosis(Mya)	21–60	?	?	?	?	45–65	24–34
**Agriculture**							
Mode of nesting	Xylem tunnels & chambers	Phloem tunnels & chambers	Xylem tunnels	Plant galls	Xylem tunnels	Subterranean nests (occ. mounds)	Subterranean nests and mounds
Substrate for fungi	Surrounding wood	Surrounding phloem (and wood)	Surrounding wood	Surrounding plant tissue	Surrounding wood	Collected plant material (twigs, caterpillar feces, leaf litter, flowers, fruits, fresh leaves)	Collected plant material (dry leaf litter, twigs, wood)
Mode of agriculture^2^	Advanced	Primitive (possibly advanced in *Dendroctonus*)	Primitive	?	Primitive	Advanced	Advanced
**Enzymatic profile**							
Fungus garden (incl. microbial community)	xylem degrading saprotrophism and bionecrotrophism^5^	bionecrotrophism of phloem	?	?	xylem degrading saprotrophism	Saprotrophism (saprobic and biotrophic in leaf-cutting ants)	Saprotrophism (plant cell-wall degrading)
Fungus acquired enzymes^3^	Possible^5^	?	?	?	Present	Present	Present
**Mode of feeding**^**4**^							
Adults	Mycetophagy	Phloeomycetophagy	No food	Plant sap	No food	Mycetophagy, (plant material)	Mycetophagy, (plant material)
Larvae	Mycetophagy (Xylomycetophagy^6^)	Phloeomycetophagy	Xylomycetophagy	Mycetophagy	Xylomycetophagy	Mycetophagy	Mycetophagy

^1^ Here we only refer to bark beetles in nutritional symbioses with fungi and omit species only feeding on phloem.

^2^ Primitive fungiculture is defined by only dispersal and seeding of fungi; advanced fungiculture additionally involves the active care of fungal crops (cf. [9]).

^3^ Evidence for fungus acquired enzymes that are active in the insect gut or fecal exudates [13,62].

^4^ Distinctions originating from the scolytine beetle literature e.g. [20]: Mycetophagy = eating fungal mycelium, fruiting bodies or specific fungal structures, Phloeomycethophagy = eating phloem and fungal biomass, Xylomycetophagy = eating xylem and fungal biomass.

^5^ Reference: this study.

^6^ Only in larvae of the genus *Xyleborinus* and probably *Xylosandrus*[21,32].

## Conclusions

Despite differences in the type of substrate used to cultivate symbiotic fungi, a striking, but perhaps not surprising commonality between the major insect fungus-growing systems is the direct or indirect use of a similar set of fungal carbohydrate active enzymes to utilize recalcitrant plant material as a stable food source (Table 1). Plant cell-wall degrading xylanases, pectinases and to a lesser degree cellulases dominate the enzymatic profiles in all cases, although inherent variation between fungus-growing systems are certainly present at the level of specific enzymes. Endogenously produced cellulase enzymes are not common among arthropods [63] (for a contrasting view see [58]), which indicate that the provision of essential carbohydrate active enzymes by microbes facilitates fungus farming.

Feeding activity of *X. saxesenii* larvae not only benefits other group members by creating more space for the ambrosia fungus to form ambrosial layers on the gallery walls, but here we show that it also enhances wood degradation and nutrient cycling. Predigested larval feces, which contains small woody particles and probably also enzymes, is smeared on gallery walls after defecation [21,33]. The wood particles in this fecal inoculum may be further degraded and nitrogenous excretions recycled by the ambrosia fungi [64]. This may in turn explain the positive effect of larval numbers on group productivity in *X. saxesenii*[21], and demonstrates a synergism between age groups that prevents competition for fungal food, because adults and larvae feed differently and apparently use a complementary set of enzymes. The differences in enzyme profiles of *X. saxesenii* larvae and adults are interesting for understanding the social system of this species. *X. saxesenii* is the only primitively eusocial ambrosia beetle described (characterized by overlapping offspring generations, cooperative brood care and reproductive division of labor) and similarly to the obligatorily eusocial ants, bees and termites exhibit division of labor not only between the sexes, but most importantly also between larval and adult offspring [21]. Differential enzyme activity therefore adds an additional layer of complexity to the behavioural division of labour between adults and larvae. Production of extra enzymes and nutrients by larvae (and their trophallaxis to adults) has been reported from other social insects, such as ants and wasps [65-67], and larvae of the leaf-cutting ant *Acromyrmex subterraneous* have even been denoted the “digestive caste” of the colony based on the extensive enzymatic machinery detected in their gut lumen [68]. Holometabolous insects dramatically restructure morphology and physiology during metamorphosis and phenotypes of larval and adult stages thus represent distinct developmental and evolvable modules compared with the highly correlated life stages of insects with “incomplete” metamorphosis (Hemimetabola) [69]. Because of this predisposition we propose that larvae in holometabolous insect societies may play a much more important role in resource utilization than is currently recognized.

## Materials and methods

### Laboratory breeding

*X. saxesenii* adult females were collected in the Spilwald forest (560 m asl; 46°95’, 7°31’) close to Bern, Switzerland in January 2010, by dissection of galleries from stumps of beech trees (*Fagus sylvatica*) that had been cut about a year earlier. From these galleries adult *X. saxesenii* females were brought to the laboratory and placed individually in ~15 mL plastic tubes filled with a sterile nutrient-enriched beech saw-dust media solidified with agar as previously described [50]. *X. saxesenii* galleries typically consist of a straight entrance tunnel dug perpendicular into the media for about 2–5 cm where it reaches a flat brood chamber of 2–3 cm^2^ and a height of 1 mm (Figure 1A, [Additional file 1: Figure S3]). Three distinct gallery compartments - entrance tunnel, brood chamber and gallery dump material – may be discerned both in laboratory galleries in artificial media and field galleries constructed in wood. *X. saxesenii* is obligately sib-mating (inbreeding) within the natal nest and dispersing females vertical transmit the associated mutualistic symbionts in mycetangia [21]. Dispersing adults can be collected from the surface of the media and thus enables breeding of consecutive generations in the laboratory.

Galleries used in this study were from the 5^th^ laboratory generation. Sampling from laboratory ambrosia beetle galleries is preferable to sampling from field galleries, because this allows (i) to control the age of the galleries (and thereby changes in the fungal composition) and (ii) to monitor fungal diversity and the number of beetles and their behavior. Laboratory breeding of ambrosia beetles also has disadvantages, because symbiont composition may differ between laboratory and field galleries. Although it is unlikely that new microbes have invaded the system, because of the highly specialized vertical transmission of the primary mutualists in beetle mycetangia and relatively few other secondary microbes on the integument [14], it is possible that relative composition of symbionts has changed in response to the different conditions within the laboratory. Changes are probably negligible, however, because ambrosia beetles have up to now (March 2012) been reared for ten successive generations within the laboratory and major changes in gallery productivity across generations are absent, which indicates that the abundance of the primary symbionts in the microbiome is unaffected by long-term laboratory rearing [unpublished data (Biedermann PHW)].

### Sampling and protein extraction

In this study we collected samples from laboratory maintained galleries at three particular time points during gallery development: (A) At day 45 after gallery foundation (= *age45*) when few adults, but many 1^st^ and 2^nd^/3^rd^ instar larvae are present in the gallery and the microbiome is dominated by the *Raffaelea sulfurea* symbiont [24]. (B) At day 62 after gallery foundation (= *age62*) when few immature brood, but many more adults that are just starting to disperse are present in the gallery. The microbiome has changed and is no longer completely dominated by *R. sulfurea,* but contains also a mixture of several saprobes (e.g. *Paecilomyces* and *Penicillium* species; [24]. (C) At day 87 after gallery foundation (= *age87*) when production of new brood has ceased and almost all adult offspring has left the gallery. The microbiome is dominated by a few saprobic species, which are probably of little nutritional value to the beetles [24].

When sampling, we removed the solid agar-sawdust based medium containing the beetle galleries from the plastic tube in a single large piece and subsequently dissected it using a scalpel and forceps. Thirty mg (wet weight) of gallery material from the three distinct gallery compartments: (I) entrance tunnel, (II) brood chamber, and (III) expelled material from the gallery dump were collected and weighed on an electronic scale (0.0001 g precision). Total proteins were extracted from each sample, put in an Eppendorf tube filled with 260 μl ddH_2_0 and 0.1% Tween20, and ground with a small plastic pestle. Tween20 was added to the extraction water to keep enzymes in suspension [70]. Samples were vortexed, centrifuged at 15.000 g for 15 min at 4 °C and enzyme activity of the supernatant fraction was immediately measured to minimize internal proteolysis. In total 11, 15 and 8 galleries from *age45*, *age62* and *age87*, respectively, were used giving a total sample size of 34 galleries times three compartments (11 gallery dump samples had to be discarded as there was not enough material). In addition, we counted all individuals (1^st^ instar, 2^nd^/3^rd^ instar larvae, and adults) present within a gallery at that time.

In addition to sampling gallery material we also measured individuals for enzyme activity. First instar larvae, 2^nd^/3^rd^ instar larvae and adult beetles were collected at *age45*, when all developmental stages of *X. saxesenii* were present within galleries. All individuals were surface sterilized once in bleach and once in 96% alcohol. Three adults, four 2^nd^/3^rd^ instar larvae and twelve 1^st^ instar larvae were combined per sample to standardize the amount of biological material to approximately 30 mg biomass. Thereafter, samples were grinded in 60 μl ddH_2_0 containing 0.1% Tween20, vortexed, centrifuged (see above) and immediately used for enzyme activity measurements.

### Enzyme activity measurements

Enzyme activity was assayed with Azurine-Crosslinked (AZCL) polysaccharides that are purified polysaccharides cross-linked with a blue dye to form a water insoluble substrate, which is commercially available from Megazyme^©^ (Bray, Ireland) in the form of a powder (Table 2). Assay plates were prepared as previously described [71,72] with a medium consisting of 1% agarose, 23 mM phosphoric acid, 23 mM acetic acid and 23 mM boric acid, mixed and adjusted to pH = 6. The medium was heated using a microwave to melt the agarose. When the medium had cooled to 65 °C, 0.1% weight/volume AZCL substrate wetted in 96% ethanol was added. The medium was then poured into Petri dishes and allowed to solidify. Thereafter, we made five wells (~4 mm^2^) per plate using a cut-off pipette tip, applied 15 μl supernatant of each protein enzyme extract per well, and incubated the plates at room temperature (ca. 21 °C) in the dark. After 24 h all plates were photographed for quantifying the area of the blue halo surrounding each well with image analysis software (ImageJ ver. 1.37v, W. Rasband, http://rsb.info.nih.gov/ij/). A positive enzyme reaction lead to the release of dyed water soluble fragments into the agarose medium and the area of blue coloration is thus a quantitative measure for enzyme activity that can be compared between samples [71-73], although it does not provide absolute values of enzyme activity [74]. 13 AZCL substrates were tested for enzyme activity (Table 2), except for the larval and adult beetle samples that were only tested for *endo-*β-1,4-glucanase, *endo-*β-1,3(4)-glucanase, *endo-*β-1,4-xylanase, and *endo-*protease activity because of insufficient extracts to test for all 13 substrates. A pilot study showed no activity of either gallery, beetle or larval extracts against the substrates AZCL-pullulan, AZCL-chitosan, AZCL-curdlan, and AZCL-pachyman and therefore results for these substrates were not shown here.

**Table 2:** Insoluble chromogenic substrates used to test for enzyme activity and the specific type of enzymes measured

**Substrate**	**Enzyme**
**Cellulose**	
AZCL-HE-Cellulose	cellulase (*endo*-β-1,4-glucanase)
AZCL-Barley β-Glucan	cellulase (*endo*-β-1,3(4)-glucanase)
AZCL-Xyloglucan	*endo*-β-1,4-xyloglucanase
**Hemicellulose**	
AZCL-Xylan	*endo*-β-1,4-xylanase
AZCL-Arabinoxylan	*endo*-β-1,4-xylanase
AZCL-Dextran	*endo*-α-1,6-dextranase
**Pectin**	
AZCL-Debranched Arabinan	*endo*-α-1,5-arabinase
AZCL-Rhamnogalacturonan	rhamnogalacturonanase
AZCL-Galactomannan	*endo*-β-1,4-mannanase
AZCL-Galactan	*endo*-β-1,4-galactanase
**Protein**	
AZCL-Casein	*endo*-protease
AZCL-Collagen	*endo*-protease
**Starch**	
AZCL-Amylose	α-amylase

*AZCL* = Azurine cross-linked polysaccharides (Megazyme^©^, Bray, Ireland).

### Data analysis

Enzyme activity of the gallery data were ‘log + 1’ transformed to normalize the data. Enzyme activity were analyzed for each substrate in separate mixed linear models with (A) the three factorial variables (i) gallery compartment (three levels: ‘entrance’, ‘brood chamber’ and ‘gallery dump’), (ii) the interaction between gallery compartment and age of the gallery (three levels: *age45*, *age62* and *age87*), (iii) the interaction between gallery compartment and beetle composition (three levels: ‘adult beetles and immatures present’, ‘only immatures present’ and ‘no beetles or larvae present’) and (B) the continuous variables (i) total number of adults and (ii) total number of larvae. All variables were included as fixed effects. Each gallery was assigned a code that was included as a random factor in all models because entrance, brood chamber and gallery dump samples from the same gallery are not independent measurements. Model estimation was performed with Maximum Likelihood using the lme function implemented in R [75] and each variable was evaluated by ANOVA analysis of log-likelihood scores using a step-wise model reduction scheme. Specific means were compared with Tukey’s multiple comparisons of the final model. The correlation between a particular enzyme activity and the three sample gallery compartments were analyzed using partial least square regression of a matrix consisting of three x-variables (sample location: entrance tunnel, brood chamber and gallery dump) and 13 y-variables (enzyme activity for each substrate screened) using the R package pls [76] [see Additional file 1.

## **Competing interests**

The authors declare that they have no competing interests.

## Authors’ contributions

HHDFL and PHWB designed the study. PHWB collected and maintained laboratory beetle galleries. HHDFL and PHWB conducted enzyme measurements and HHDFL analyzed the data. HHDFL and PHWB wrote the manuscript in collaboration. All authors read and approved the final manuscript.

## Authors’ information

HHDFL is postdoctoral fellow at the Microbial Ecology Group at Lund University, Sweden, and studies the ecology and evolution of mutualistic systems. PHWB is Ph.D. student at the Department of Behavioural Ecology at University of Bern, Switzerland. PHWB studies the evolutionary mechanisms of social and mutualistic interactions.

## Supplementary Material

Additional file 1 Supplementary Online Material. Additional figures supporting the data analysis. Click here for file
