# Supplementary material for: Patterns of functional enzyme activity in fungus farming ambrosia beetles
Source: Front Zool. 2012 Jun 6;9:13. doi: 10.1186/1742-9994-9-13 (PMC3502098; doi:10.1186/1742-9994-9-13)

SUPPLEMENTARY ONLINE MATERIAL

Patterns of functional enzyme activity in fungus farming ambrosia beetles

Henrik H. De Fine Licht[1*] and Peter H. W. Biedermann[2]

[1]Microbial Ecology Group, Department of Biology, Lund University, Ecology Building, Solvegatan 37, SE-223 62 Lund, Sweden, Email: Henrik.de_fine_licht@biol.lu.se.

[2]Department of Behavioural Ecology, Institute of Ecology & Evolution, University of Berne, Baltzerstrasse 6, CH-3012 Bern, Switzerland, Email: peter.biedermann@iee.unibe.ch.

[*]Corresponding Author.

Figure S1.

X. saxesenii laboratory gallery enzyme activity (mean+SE) against 13 specific carbohydrate substrates measured at 45, 62 and 87 days, respectively, after gallery foundation by a single mated female. The same data is presented as a heatmap in figure 1 in the manuscript. For ease of interpretation the polysaccharide substrates are divided into five major groups based on enzyme activity: Cellulases – cellulose, beta-glucan, xyloglucan; Cross-linking glycans – xylan, arabinoxylan, dextran; pectinases – galactomannan, galactan, rhamnogalacturonan, debranched arabinan; Proteases – casein, collagen; and Amylase – amylose. Different letters above horizontal columns indicate significant different means in post-hoc Tukey's tests. Ns = non-significant, k-w = Kruskal-Wallis test with a * denoting significance at $p < 0.05$. Sample sizes for entrance tunnel, brood chamber, and gallery dump respectively are: n = 16, n = 16, n = 11 at age 45, n = 11, n = 13, n = 5 at age 62, and n = 8, n = 8, n = 2 at age 87.

Figure S2.

Partial least square regression of gallery enzyme activity at age 45 (**A**), age 62 (**B**), and age 87 (**C**). X loadings are gallery sampling points (brood chamber, entrance tunnel, and gallery dump); Y loadings are specific enzyme activity: cellulos = HE-cellulose (cellulase), betaglu = *β*-glucan (cellulase / ß-glucanase), xyloglu = xyloglucan (cellulase), xylan = *endo*-xylanase, axyl = arabinoxylan (*endo*-xylanase), galman = galactomannan (*endo*-1,4-ß-mannanase), galac = galactan

(*endo*-1,4-ß-galactanase), debarab = debranched arabinan (*endo*-arabinase), casein = *endo*-protease, amyl = amylose (α-amylase). The closer an enzyme substrate is to one of the three samples, the higher the particular enzyme activity is in that sample.

Figure S3.

Picture of a *X. saxesenii* brood chamber (top) with 1st and 2nd/3rd instar larvae, pupae and teneral females surrounded by lush mycelial growth of the symbiont *Raffaelea sulfurea* and close-up of two adult females (bottom) inside a gallery with mites. Both pictures are form laboratory galleries and the top picture is taken through the plastic tube containing the gallery. © P. H. W.Biedermann.

Figure S1

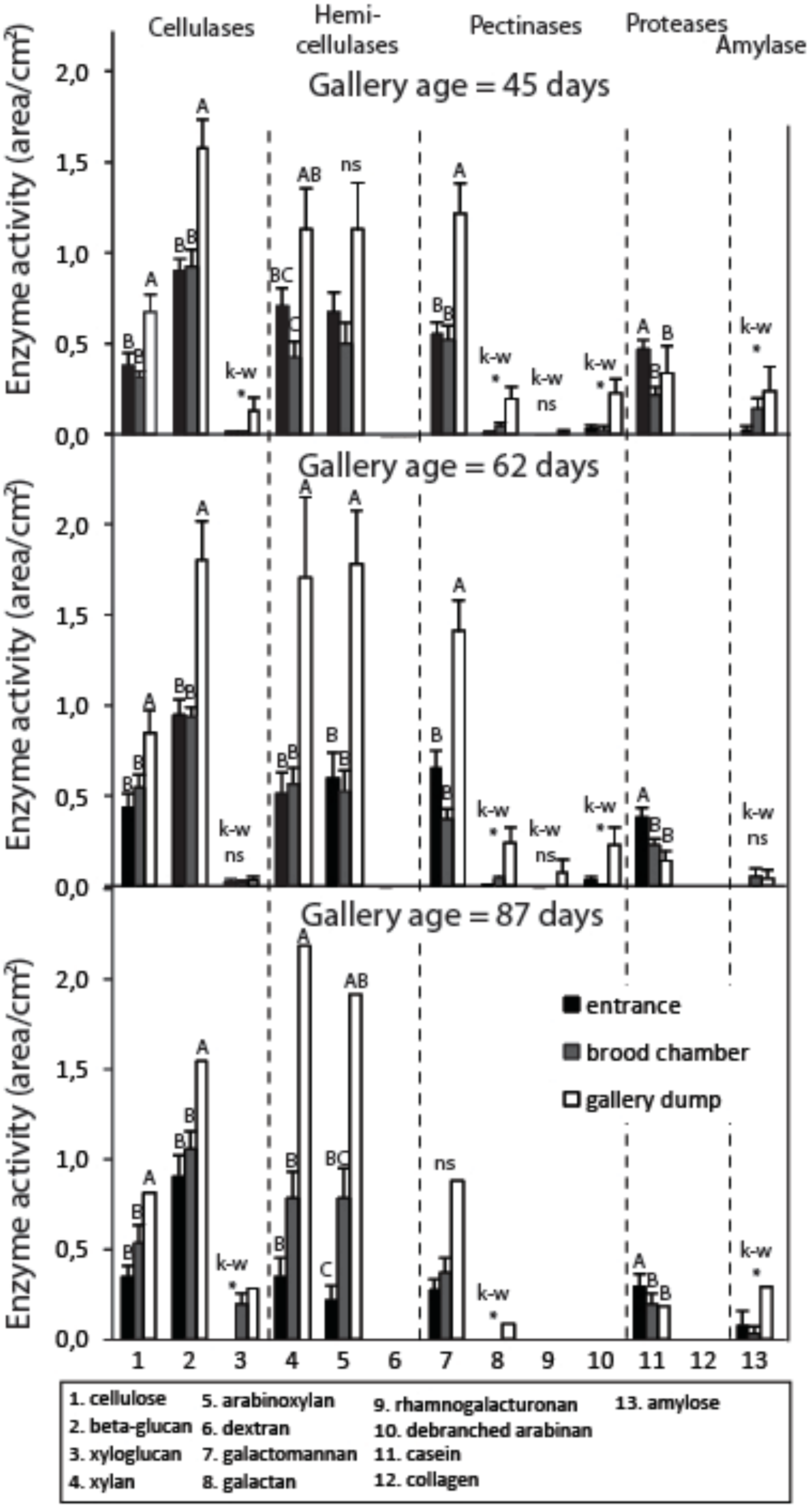

Cellulases
Hemi-cellulases
Pectinases
Proteases
Amylase
Gallery age = 45 days
Gallery age = 62 days
Gallery age = 87 days
Enzyme activity (area/cm²)
entrance
brood chamber
gallery dump
1. cellulose
2. beta-glucan
3. xyloglucan
4. xylan
5. arabinoxylan
6. dextran
7. galactomannan
8. galactan
9. rhamnogalacturonan
10. debranched arabinan
11. casein
12. collagen
13. amylose

Figure S2A

Gallery age = 45

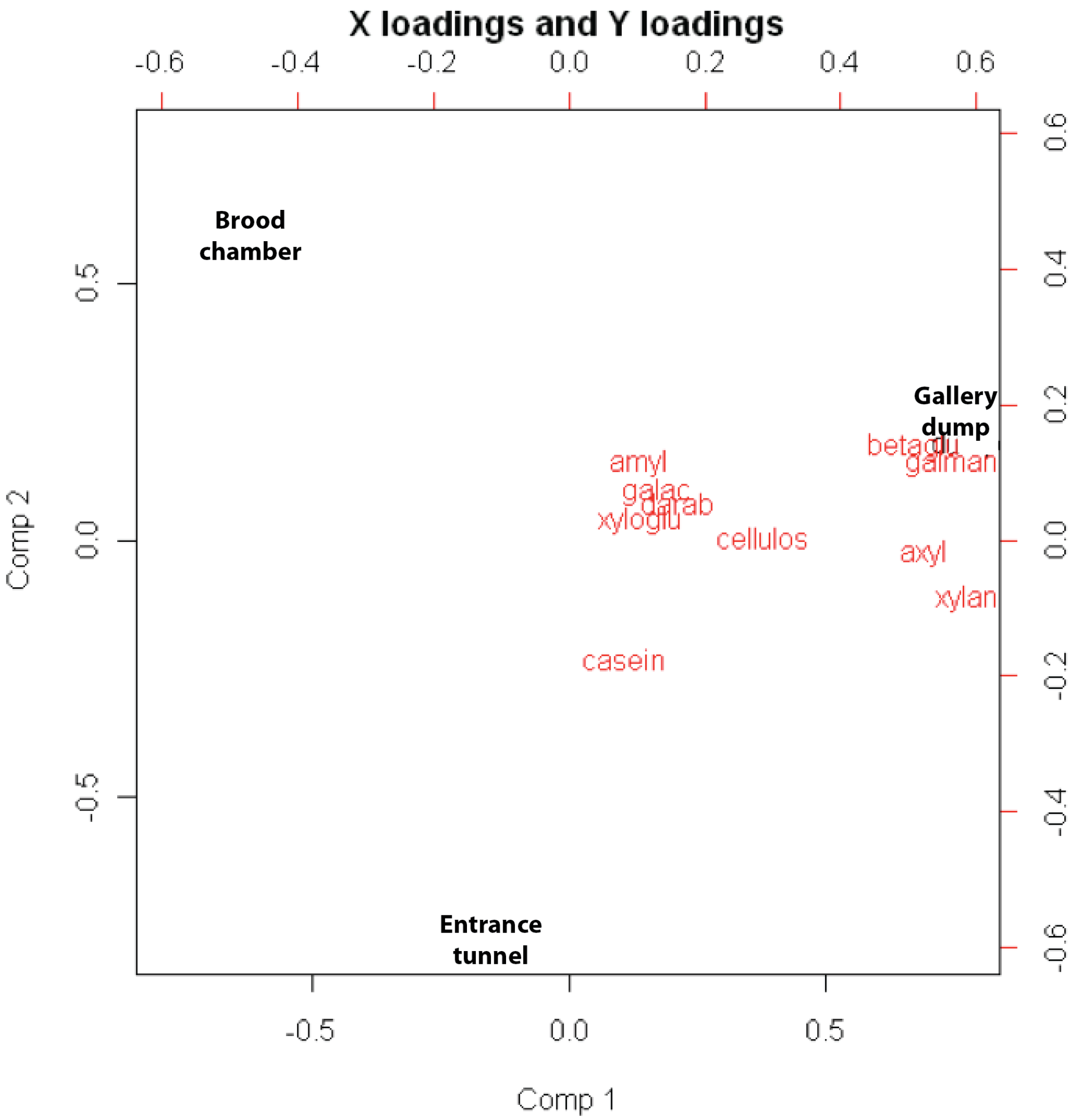

Figure S2B

Gallery age = 62

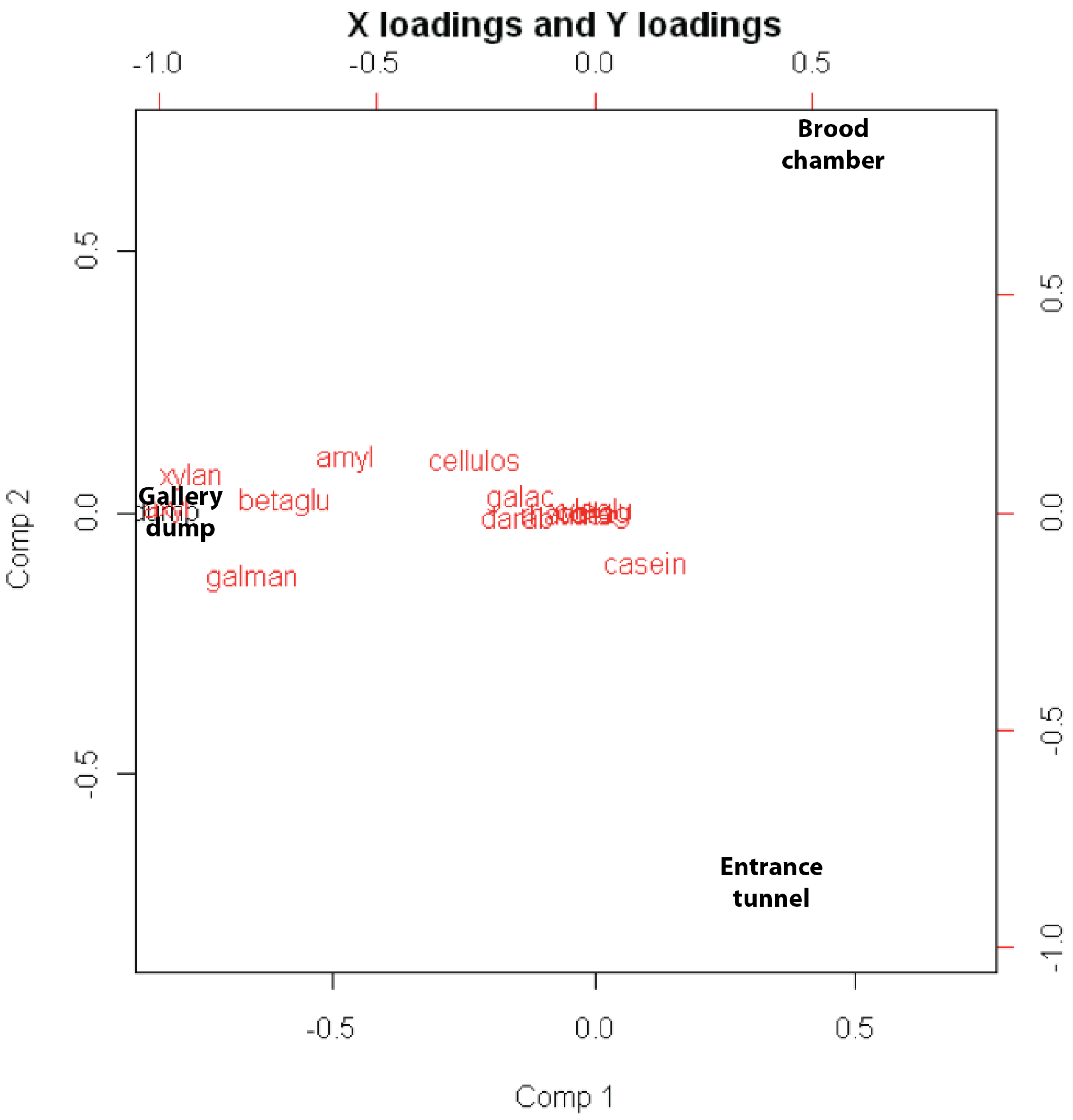

Figure S2C

Gallery age = 87

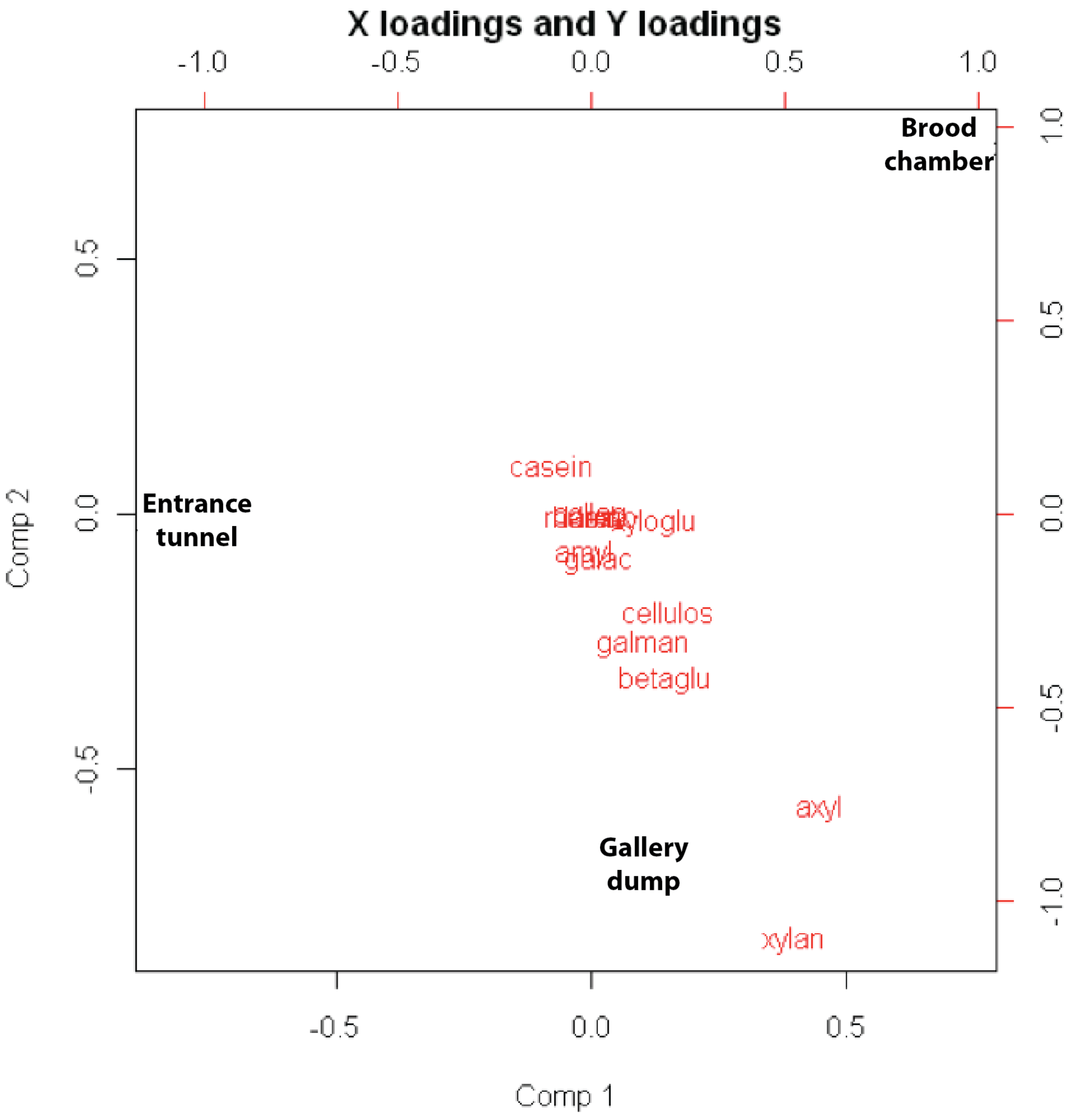

Figure S3

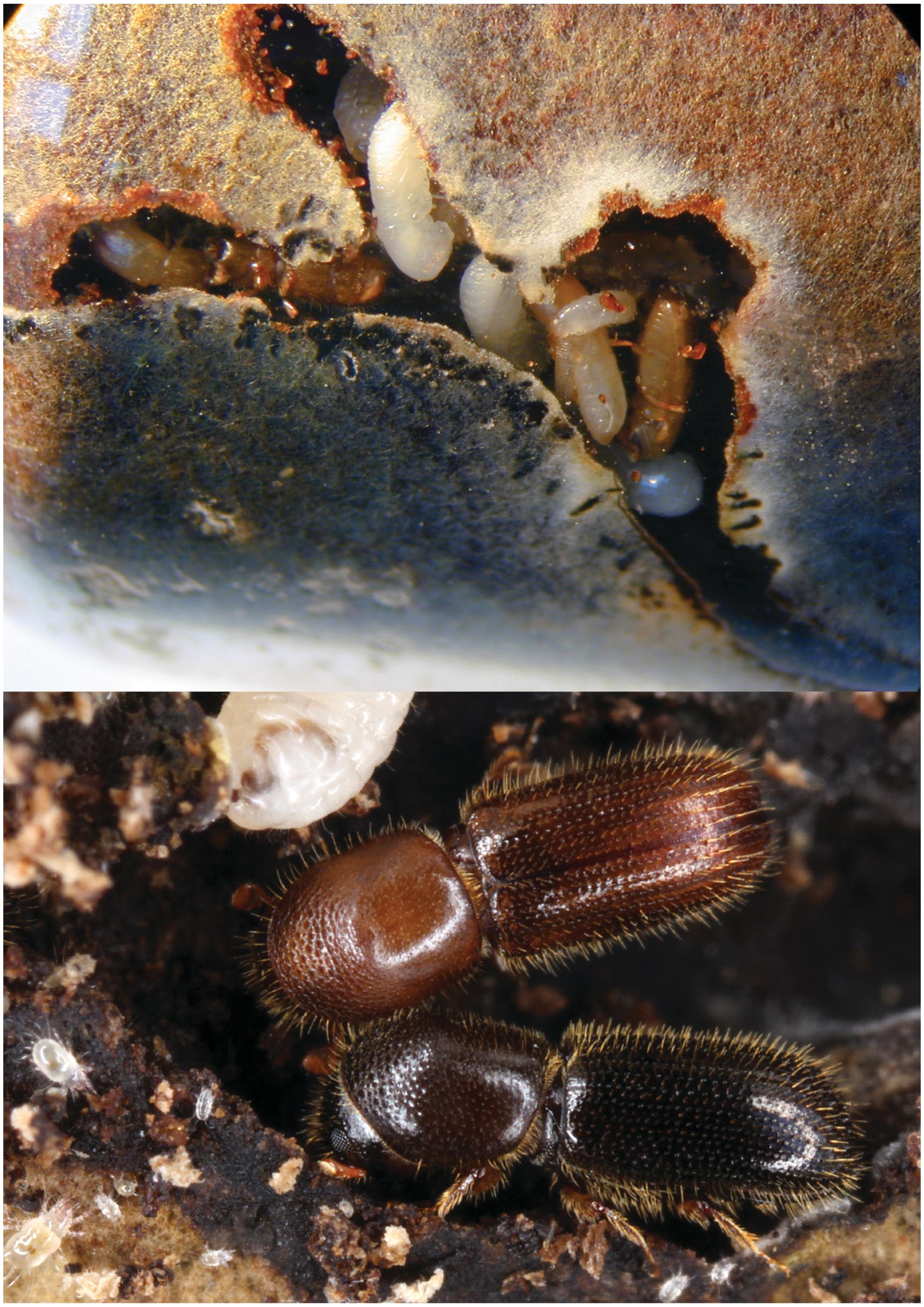

Supplement: Additional file 1 — Supplementary Online Material. Additional figures supporting the data analysis. [file 1742-9994-9-13-S1.pdf]
